# Climate factors influence seasonal influenza activity in Bangkok, Thailand

**DOI:** 10.1371/journal.pone.0239729

**Published:** 2020-09-29

**Authors:** Nungruthai Suntronwong, Preeyaporn Vichaiwattana, Sirapa Klinfueng, Sumeth Korkong, Thanunrat Thongmee, Sompong Vongpunsawad, Yong Poovorawan

**Affiliations:** Center of Excellence in Clinical Virology, Department of Pediatrics, Faculty of Medicine, Chulalongkorn University, Bangkok, Thailand; Columbia University, UNITED STATES

## Abstract

Yearly increase in influenza activity is associated with cold and dry winter in the temperate regions, while influenza patterns in tropical countries vary significantly by regional climates and geographic locations. To examine the association between influenza activity in Thailand and local climate factors including temperature, relative humidity, and rainfall, we analyzed the influenza surveillance data from January 2010 to December 2018 obtained from a large private hospital in Bangkok. We found that approximately one in five influenza-like illness samples (21.6% or 6,678/30,852) tested positive for influenza virus. Influenza virus typing showed that 34.2% were influenza A(H1N1)pdm09, 46.0% were influenza A(H3N2), and 19.8% were influenza B virus. There were two seasonal waves of increased influenza activity. Peak influenza A(H1N1)pdm09 activity occurred in February and again in August, while influenza A(H3N2) and influenza B viruses were primarily detected in August and September. Time series analysis suggests that increased relative humidity was significantly associated with increased influenza activity in Bangkok. Months with peak influenza activity generally followed the most humid months of the year. We performed the seasonal autoregressive integrated moving average (SARIMA) multivariate analysis of all influenza activity on the 2011 to 2017 data to predict the influenza activity for 2018. The resulting model closely resembled the actual observed overall influenza detected that year. Consequently, the ability to predict seasonal pattern of influenza in a large tropical city such as Bangkok may enable better public health planning and underscores the importance of annual influenza vaccination prior to the rainy season.

## Introduction

Influenza virus infection is a major cause of acute respiratory disease and contributes to significant morbidity and mortality each year [1]. Seasonal pattern of influenza varies throughout the temperate, tropical and sub-tropical regions of the world [2]. In the temperate regions of both northern and southern hemisphere, influenza activity peaks during the cold winter months and is linked to increased person-to-person transmission in indoor close-contact settings and low absolute humidity [3–8]. In tropical regions, different patterns of influenza activity are observed [9, 10]. Inter-tropical belt region shows highly heterogeneous timing of influenza epidemics in which some countries have an annual peak coinciding with rainy season, biannual peaks of influenza activity, or no distinct seasonality at all.

Previous studies have examined the meteorological factors conducive to influenza transmission in different parts of the world. In temperate regions, a study in the U.K. suggests that both influenza A and B viruses preferred low temperatures and dew points [11], while a study in Canada observed that increased influenza A virus detection was associated with increased relative humidity and low temperature [12]. Results from studies in tropical regions, however, are inconsistent depending on the location and the study period. In the tropical Republic of Côte d’Ivoire, rainfall was a good predictor of influenza activity [13], while in Uganda, influenza A(H1N1) but not influenza A(H3N2) and B activity was associated with lower precipitation [14]. In Central America, the overall influenza activity was consistently associated with increased specific humidity, but temperature and the amount of rainfall were associated with influenza activity in only certain locations [15].

Influenza pattern in tropical Southeast Asia has been less well-studied compared to that in industrialized countries. Although influenza outbreaks fluctuate seasonally, major influenza activity often coincides with the rainy season in Cambodia, Laos PDR, Myanmar and the Philippines [16–19]. Thailand experiences primary influenza activity during the rainy season, with an additional secondary peak of less magnitude at the beginning of the year [17, 20]. Meanwhile, some studies in Indonesia, Singapore, and Vietnam reported no consistent pattern of influenza peak [17, 21, 22]. Circulating influenza virus strains in Southeast Asia have mostly been A(H1N1)pdm09, A(H3N2), and influenza B virus [23]. Yet, few studies have examined in-depth the seasonality of influenza with local climates. It was reported that increasing temperature and humidity has been linked to increased influenza activity in the Philippines and Vietnam [2, 24]. However, neither temperature nor humidity was positively associated with influenza A and B virus in Singapore [25].

In Thailand, data from suspected influenza cases suggest that temperature and relative humidity, but not rainfall, correlated with the overall influenza activity in central Thailand [26]. In that study, information regarding individual influenza virus (sub)types were not available, and the prediction models for influenza activity differed depending on geographical regions. It was possible that the diversity of regional climates, such as in the higher elevations of the north, the sea-bound southern peninsula, and the low elevation of the central plain, may also complicate the generalization of climate influence on influenza activity within the country. Bangkok is situated in central Thailand adjacent to the Gulf of Thailand where the prevailing hot and humid weather is disrupted by the cooler and drier air from the north a few weeks each year (typically between December and February) [27]. Since fluctuating climate factors may influence the seasonality of influenza activity in and around metropolitan Bangkok, we examined the multi-year incidence of influenza virus infection and how different influenza virus (sub)types were associated with the monthly meteorological factors of temperature, relative humidity, and rainfall. Using time series analysis, we examined potential forecasting models for influenza activity. Improved understanding of the seasonal pattern of influenza virus infection for a large urban area may guide public health policies on the optimal timing of influenza vaccination in the country.

## Materials and methods

### Ethical approval

The study was approved by the Institutional Review Board of the Faculty of Medicine of Chulalongkorn University (IRB No. 127/61). Consent was not obtained because the data were analyzed anonymously.

### Laboratory testing of influenza virus

From January 2010 to December 2018, 30,852 nasopharyngeal or throat swab samples from patients with influenza-like illness (ILI) from Bangpakok 9 International Hospital (latitude 13.75°N and 100.55°E longitude) in Bangkok were analyzed as part of an ongoing influenza surveillance [23, 28]. We defined ILI as fever >38°C accompanied by cough and/or sore throat. Respiratory specimens arrived with limited patient information and included only age and gender. Samples were tested by using real-time reverse-transcription polymerase chain reaction to identify influenza A(H1N1)pdm09, A(H3N2) and influenza B virus as previously described [29].

### Meteorological data

Data on the monthly average temperature (°C), rainfall (cm^3^), and relative humidity (%) from January 2010 to December 2018 were available from Wolfram Alpha database [27]. They were recorded at VTBD (Don Mueang International Airport) in Bangkok (latitude 13.91°N and 100.6°E longitude).

### Data analysis

Monthly incidence of influenza was compiled based on the sample collection date. Percentages of monthly influenza virus-positive samples relative to all ILI samples each month were determined. Univariate analysis of the meteorological variables including average temperature, relative humidity and rainfall was done on data between influenza-active months and months without influenza activity using Mann-Whitney U test. An influenza-active month was defined as a month in which the proportion of the confirmed influenza cases was equal to or greater than a fixed threshold of 10% [10, 30]. This was calculated by dividing the monthly confirmed influenza cases by each year’s total number of influenza virus-positive samples [31].

To examine the seasonality of influenza, we applied a generalized linear model (GLM) with Gaussian and Poisson distribution on the monthly values of climate variables and laboratory-confirmed influenza cases, respectively [32]. A seasonal model in the waveform with a period of 12 months was then established with the estimated parameters from the GLM [11, 33].

### Cross-correlation analysis and forecasting model

We initially examined the relationships between each influenza virus (sub)type and all influenza activity with respect to meteorological variables. The monthly confirmed cases of influenza A(H1N1)pdm09, influenza A(H3N2), influenza B, and all influenza viruses were considered as dependent variables. Monthly values of mean temperature, relative humidity, and accumulated rainfalls were considered as independent variables. Cross-correlation analysis was conducted to assess the relationship between virus incidence and various meteorological factors, and for relationships among influenza A(H1N1)pdm09, influenza A(H3N2), and influenza B virus with different lag times (defined as the difference between time of maximum climate factor values and time of maximum influenza incidence) of between 1 to 4 months [26].

We considered four forecast models for individual and all influenza viruses using dependent variables from influenza A(H1N1)pdm09 (model 1), influenza A(H3N2) (model 2), influenza B (model 3), and all influenza viruses (model 4). For each model, we also considered potential association among different influenza viruses. Climate factors were considered as independent variables or proxy predictors in forecasting future influenza activity. Time series analysis was conduct to identify the best-fit model for each influenza (sub)type and for all influenza viruses in two ways. A univariate time series used past data as dependent variables to predict the future, while multivariate time series used the combined data from dependent and independent variables for prediction. The forecast model used data from January 2011 to December 2017 to calibrate and estimate the parameters. The last 12 months of data were used to test the forecasting ability. Data from 2010 were excluded due to bias from the influenza pandemic of 2009.

The Auto-Regressive Integrated Moving Average (ARIMA) (p,d,q) model was used in this study. The parameter p represents maximum lag in the auto-regression (AR). The non-seasonal differencing (d) is the order of integration (I) and represents the number of differences required to make the series stationary. The parameter q of the moving average (MA) represents the linear regression of the output value on the time series when compared with the current and previous terms [34]. Additionally, seasonal autoregressive integrated moving average model SARIMA (p,d,q)(P,D,Q)_s_ was also used. The parameters P, D, Q, and s represent the order of seasonal AR, the degree of seasonal differencing, the seasonal moving average, and the season’s length, respectively [35].

Forecasting incidence by using ARIMA model involved four steps. First, we used the augmented Dickey-Fuller (ADF) and the Kwiatkowski-Phillips Schmidt-Shin (KPSS) tests to assess whether the individual time series was stationary. The log-transformation and/or differencing was applied to render the data stationary when necessary. Second, autocorrelation function (ACF) and partial autocorrelation function (PACF) were applied to estimate p and q based on the cutoff time lag. The P and Q order was obtained from ACF and PACF peak at time s lags. Third, residual diagnosis and Ljung-Box (modified Box-Pierce) test was used to identify the possibility of the model and to diagnose white-noise. Finally, the Ljung-Box portmanteau test was applied to determine whether there was a lack-of-fit of the residuals of the time series. The null hypothesis was that the model does not exhibit lack-of-fit. It was expected that the test failed to reject the null hypothesis and that the residuals series showed white noise (p-value >0.5) [36].

The model goodness-of-fit took into account the root mean square error (RMSE), the mean absolute percentage error (MAPE), and the corrected Akaike’s Information Criterion (AICc). The lowest parameter values suggest the optimal model [37]. For multivariate model, only climate variables that provided significant lagged effect and presented the highest correlation coefficient among lag 1 up to lag 4 were considered [26, 38]. All statistical analysis was performed in SPSS version 23 (SPSS Inc., USA) and R version 3.6.0 (R Foundation, Vienna, Austria) [39]. Alpha of 0.05 denotes statistical significance.

## Results

### Influenza surveillance

**Fig 1:**
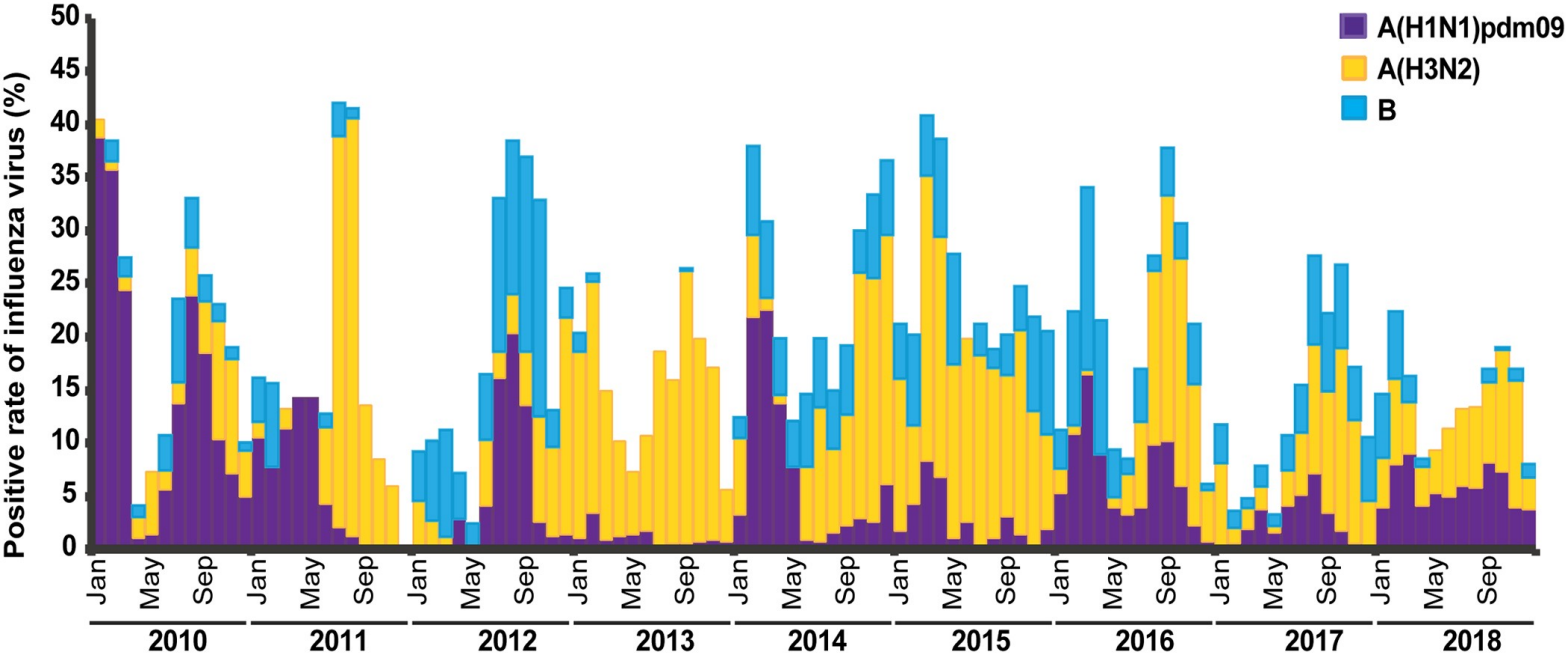
Monthly distribution of influenza activity from January 2010 to December 2018. Bar graph represents the proportion of ILI samples tested positive for seasonal influenza A(H1N1) (purple), influenza A(H3N2) (yellow), and influenza B (blue) virus each month.

In this study period, 6,678 (21.6%) of the ILI samples tested positive for influenza virus (Table 1). Of these, 80.2% (5,357/6,678) were influenza A virus and 19.8% (1,321/6,678) were influenza B virus. Influenza A virus subtypes identified were 34.2% A(H1N1)pdm09 and 46.0% A(H3N2). From 2010 to 2018, overall influenza cases generally peaked in and around August to September, which coincided with the rainy season (Fig 1). Biannual frequency of predominantly influenza A(H1N1)pdm09 virus was more apparent than that of influenza B virus.

**Table 1:** Influenza virus-positive samples and monthly average meteorological factors.

	Positives (%)	Mean (SD)^b^
**All influenza virus**	6,678 (21.6)^a^	18.93 (10.20)
A(H1N1)pdm09	2,282 (34.2)^c^	5.8 (7.19)
A/H3N2	3,075 (46.0)^c^	9.13 (8.11)
B	1,321 (19.8)^c^	4 (4.26)
**Meteorological factors**		
Temperature (°C)	-	28.73 (1.71)
Relative humidity (%)	-	73.32 (4.96)
Rainfall (cm^3^)	-	9.11 (9.46)

^a^ All influenza virus-positive samples divided by all ILI samples submitted.

^b^ Mean and standard deviation (SD) were calculated from the monthly percentage of influenza positives relative to all monthly ILI samples.

^c^ The number of specific influenza virus divided by all influenza virus-positive samples.

### Climate factors and influenza activity

**Fig 2:**
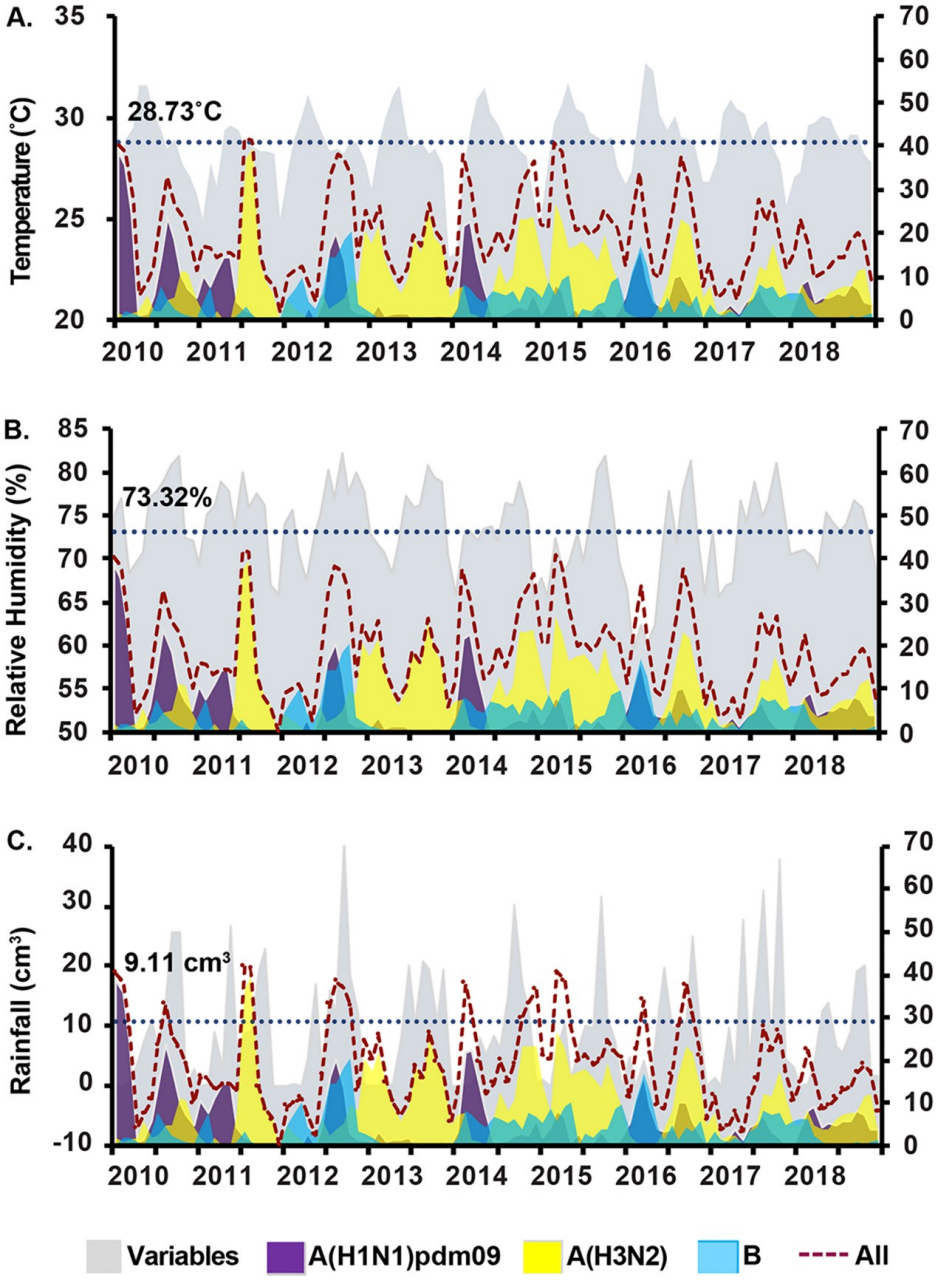
Incidence of influenza virus relative to meteorological factors. Monthly mean temperature (A), relative humidity (B), or rainfall (C) (shaded gray) are shown with the confirmed cases of influenza A(H1N1)pdm09 virus (purple), influenza A(H3N2) virus (yellow), influenza B virus (blue), and all influenza viruses (dashed line). Scale on the right Y-axis denotes the percentage of the influenza virus-positive samples each month. Dotted horizontal line indicates the overall average temperature (28.73°C), relative humidity (73.32%), and rainfall (9.11 cm^3^) over nine years (left Y-axis).

We hypothesized that certain climate factors were associated with increased influenza activity, therefore we examined monthly variations in the average temperature, relative humidity, and rainfall with respect to the monthly number of confirmed influenza virus infection. Average monthly temperature was typically highest from April to May (29–33°C) and lowest in January (23–28°C) (Fig 2A). January was also the least humid month (65–73%), while the period from September to October experienced the highest relative humidity (76–82%) and coincided with the rainy season in Bangkok (Fig 2B). Increases in the relative humidity concurred with increases in confirmed influenza A(H3N2) virus. Although the amount of rainfall varied throughout the study period, most rainfall occurred in September (10–40 cm^3^) (Fig 2C). However, the amount of rainfall did not correlate with the fluctuations of any influenza virus. When we evaluated the influence of climate factors in the months with and without substantial influenza activity, we found that only relative humidity significantly correlated with influenza virus (S1 Table).

**Fig 3:**
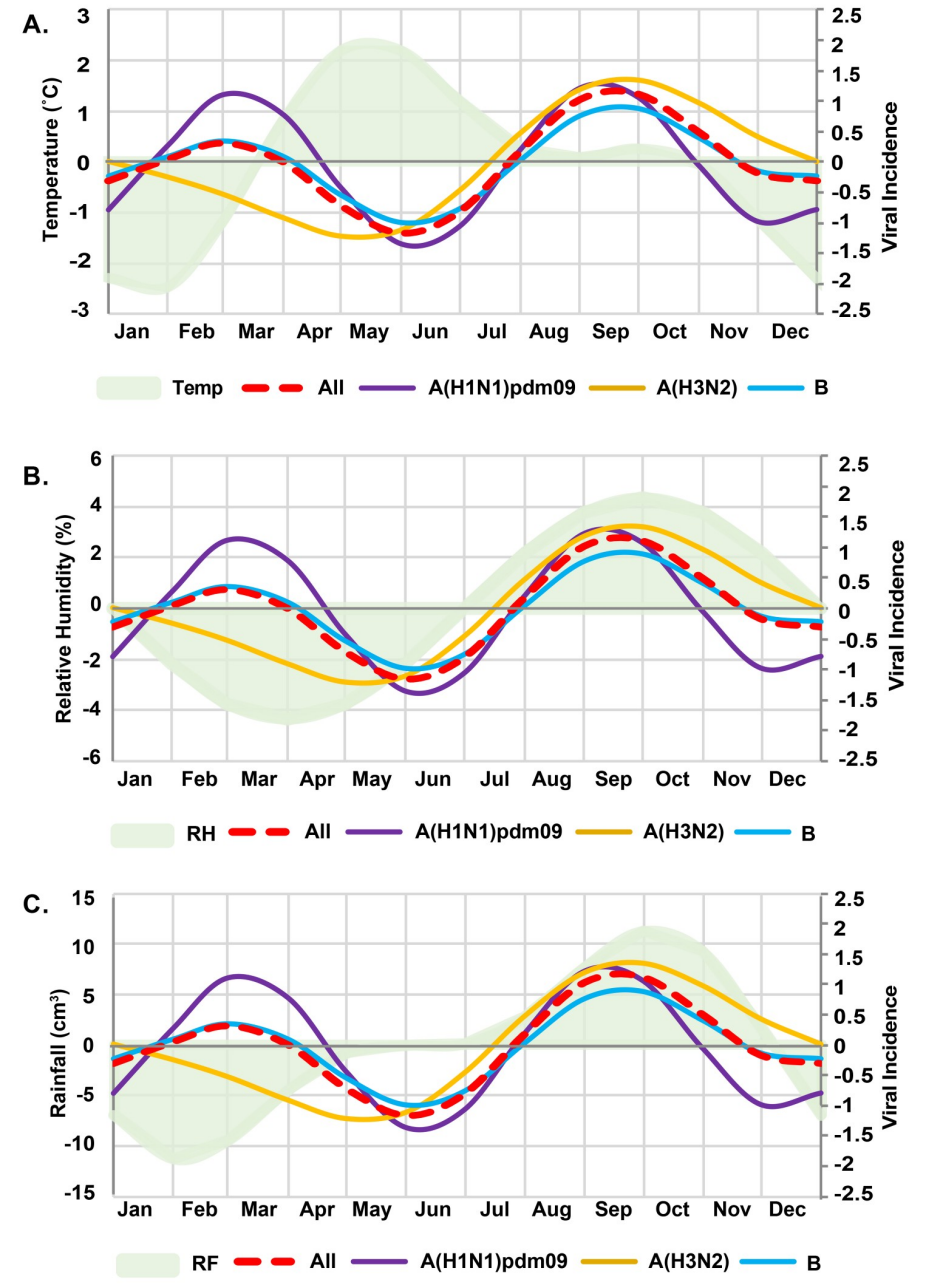
Incidence of yearly influenza and the periodic fluctuation of meteorological variables. Generalized linear models depict the incidence of influenza virus each month (colored waves) relative to the monthly mean values of climate factors (shaded). The left Y-axis represents the z-score of the (A) mean temperature (Temp), (B) relative humidity (RH), and (C) rainfall (RF). The right Y-axis represents the differences in the viral incidence for all influenza viruses (red dashed line), influenza A(H1N1)pdm09 virus (purple line), influenza A(H3N2) virus (yellow line), or influenza B virus (blue line). The highest point on the wave indicates the time of year influenza virus detection peaked.

Analysis by GLM suggests that increased influenza activity occurred twice a year (Fig 3). The first and smaller wave of increased influenza activity was in February, which corresponds to the beginning of hot and dry weather. The second and larger influenza wave occurred between August and September, which coincides with the second half the rainy season. Both periods represented increased influenza A(H1N1)pdm09 and influenza B virus activity. Meanwhile, influenza A(H3N2) activity was only associated with the second wave, which coincided with higher relative humidity and rainfall and occurred approximately 5 months after the first influenza A(H1N1)pdm09 peaked. As expected, GLM showed no association between influenza activity and temperature.

### Identifying the ARIMA(X) model

**Fig 4:**
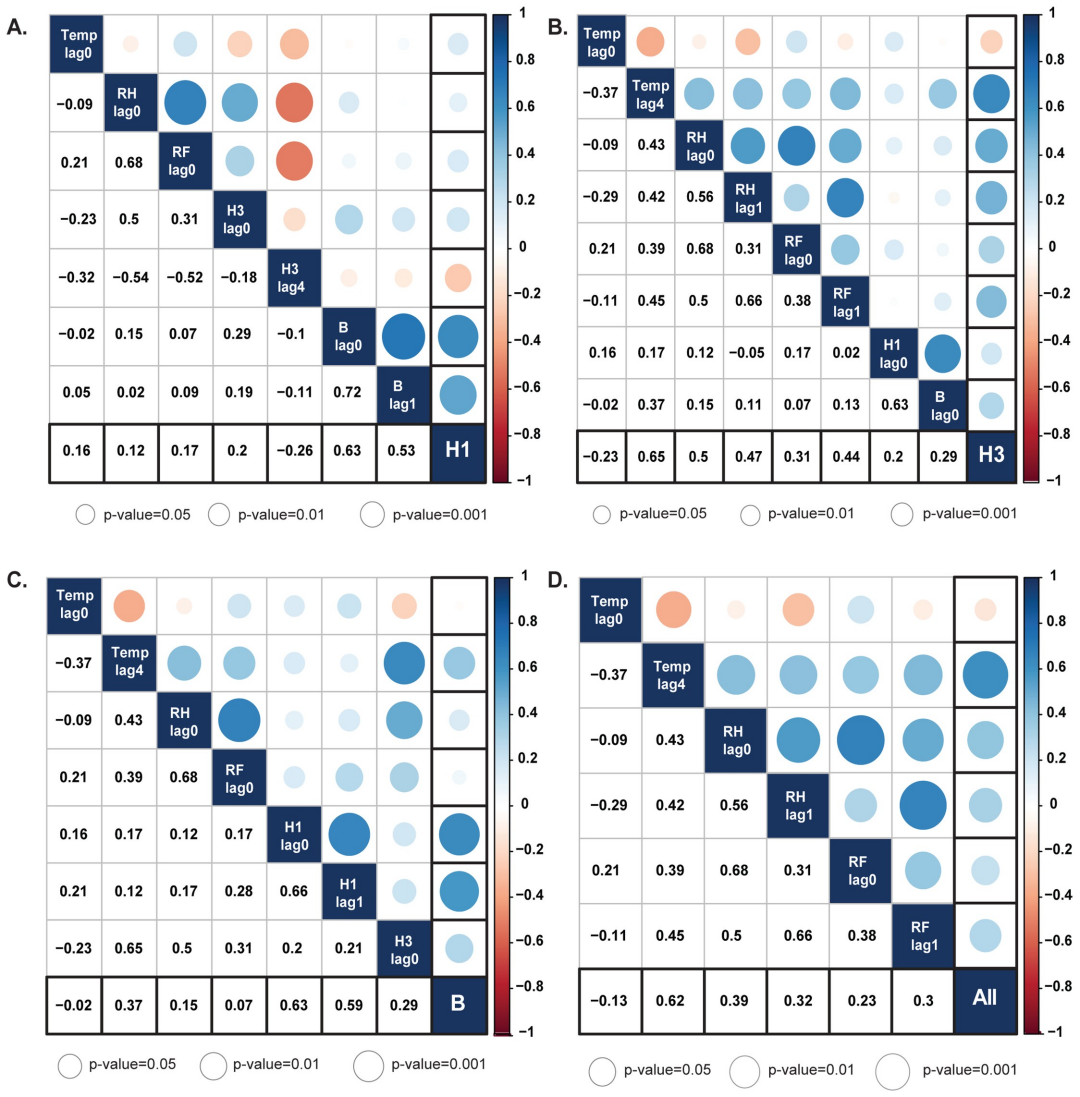
Association between meteorological factors and influenza virus. Correlation analysis of (A) influenza A(H1N1)pdm09 virus, (B) influenza A(H3N2) virus, (C) influenza B virus, and (D) all influenza viruses against climate variables and in the presence of co-circulating viruses at different lag times. Spearman’s rank cross-correlation coefficients ranged from -1 to 1. Negative value equates to negative association as indicated by red circles, and positive value equate to positive association as indicated by blue circles. Circle size represents the magnitude of the p-value. Color intensity represents the strength and weakness of the correlation (right scale). Temp, mean temperature; RH, relative humidity; RF, rainfall.

We next examined the time delay in the association between climate factors and the incidence of influenza. This was defined as the time difference in months between the peak of a climate factor and the peak of an influenza virus activity (e.g., lag 1 represents 1 month difference). We found that there was no significant association between influenza A(H1N1)pdm09 activity with any meteorological variables (Fig 4A). Influenza A(H3N2) showed a moderately positive correlation with the mean temperature of lag 4 (r = 0.65, p<0.001), and with relative humidity and rainfall of lag 1 (r = 0.47 and r = 0.44, respectively) (p<0.001) (Fig 4B). Additionally, influenza B was positively correlated with temperature at lag 4 (r = 0.37, p<0.05) (Fig 4C). To determine the correlation among influenza (sub)types, weakly negative correlation was found between influenza A(H1N1)pdm09 and influenza A(H3N2) at lag 4 (r = -0.26, p<0.05). In contrast, influenza A(H1N1)pdm09 showed strongly positive correlation with influenza B virus (r = 0.63, p<0.001). All influenza activity showed positive correlation with the mean temperature at lag 4 (r = 0.62, p<0.001), and relative humidity and rainfall at lag 1 (r = 0.32 and r = 0.3, respectively) (p<0.05) (Fig 4D).

**Fig 5:**
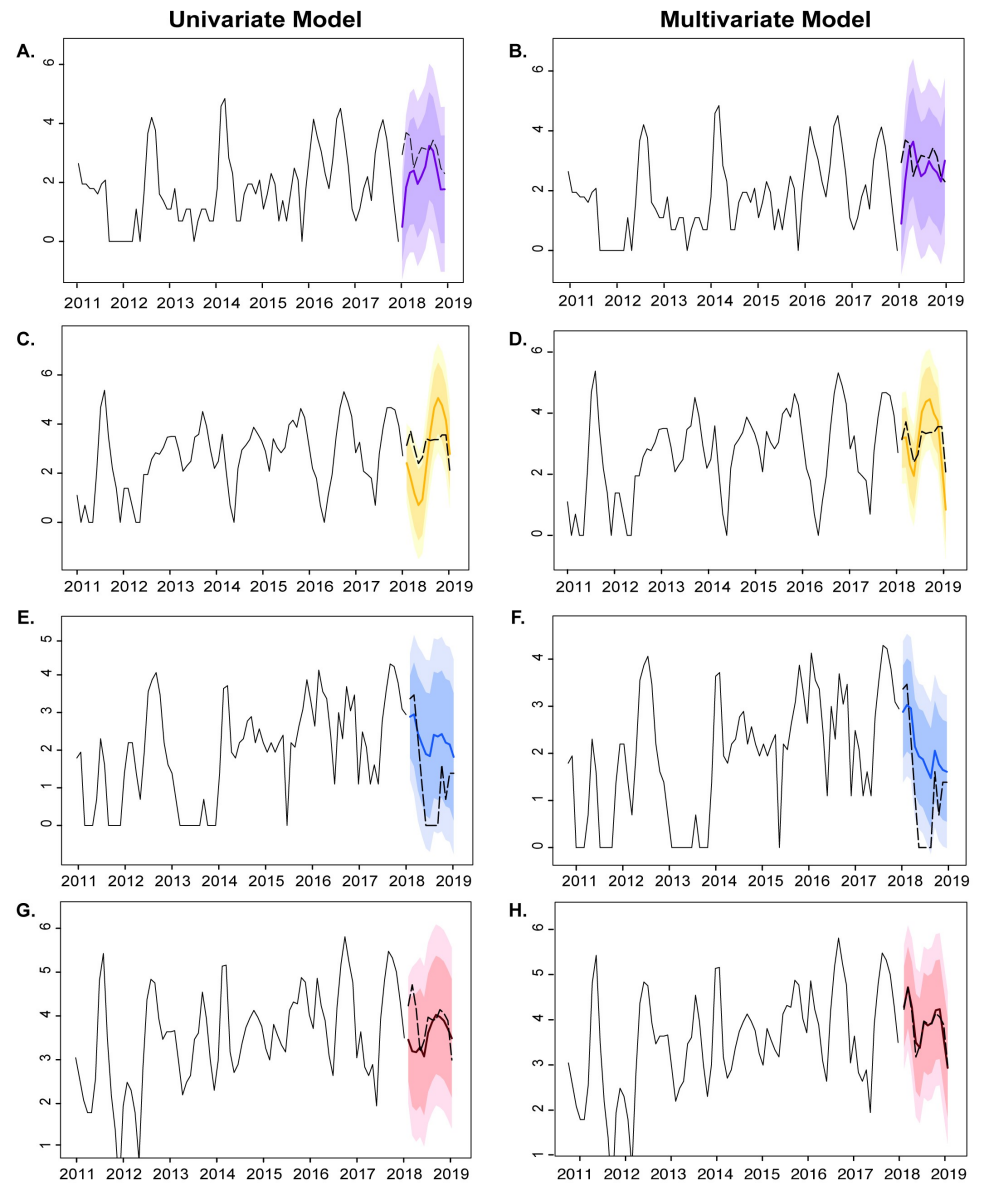
Seasonal ARIMA (p,d,q)(P, D, Q)_12_ fitted time series analysis for individual influenza (sub)type and all influenza virus activity in the different forecasting models. Time series for influenza A(H1N1)pdm09 (A, B), influenza A(H3N2) (C, D), influenza B (E, F), and all influenza virus (G, H) activities are shown in black (January 2011 to December 2017) and dashed (January 2018 to December 2018) lines. Colored lines represent the predicted seasonality trends for 2018 using univariate (left panels) or multivariate (right panels) analysis with the 90% (darker shade) and 95% (lighter shade) confidence intervals shown.

To identify the best-fitting model for the observed values, we performed the log_10_ transformation on the dependent variables to obtain a stationary time series (S1 Fig). We then computed the suitable univariate and multivariate ARIMA models. For multivariate model, variables which provided significant correlation were selected as predictors (S2 Table). The appropriate ARIMA models for individual influenza (sub)type and all influenza viruses were established (S2 and S3 Figs) by comparing the AICc values between the univariate and multivariate time series. We found that multivariate ARIMA model using both influenza incidence and climate factors provided better goodness of fit than the univariate ARIMA model, which used only influenza incidence (S3 Table). It also performed better and had reduced errors (RMSE and MAPE) than the univariate model. For influenza A(H1N1)pdm09, the SARIMA(2,1,1)(0,1,1)_12_ of multivariate time series gave a better forecast (RMSE = 0.89, MAPE = 22.99%), while the SARIMA(1,0,2)(1,1,0)_12_ model combined with all climate factors for influenza A(H3N2) provided lowest forecasting error (RMSE = 0.77, MAPE = 22.47%). In addition, the multivariate ARIMA(3,0,3)_12_ fitted well for influenza B when combined with temperature and influenza A(H1N1)pdm09 (RMSE = 1.14, MAPE = 46.09%). The SARIMA(2,0,1)(1,0,0)_12_ with the mean temperature at lag 4, relative humidity at lag 1, and rainfalls at lag 1 was the selected multivariate model with the best forecast value, which took into account all influenza and climate factors (RMSE = 0.15, MAPE = 2.81%). Fitted graphs derived from the univariate and multivariate analysis of the time series (Fig 5) showed that the multivariate models were better able to capture the observed influenza activity during 2018 than the univariate models. Overall, the forecast model using all influenza activity and all climate factors performed better than the forecast model of individual (sub)type.

## Discussion

Influenza is prevalent throughout the year in some countries with tropical climate [6]. In Thailand, increased influenza activity appears biannually typically in the rainy (June-August) and cool (December-February) seasons [20, 23, 28]. Our multi-year study confirmed the substantial decrease in influenza activity following the relatively hot and dry month of May, which precedes the rainy season and coincides with the yearly recommended influenza vaccination period in Thailand [40, 41]. We found that the first wave of influenza activity in a typical year involves A(H1N1)pdm09, and to a lesser extent influenza B virus, both of which peaked in February and again in August-September. The second and major wave of influenza activity occurs around August-September in which all influenza viruses co-circulate. The observation that influenza A(H1N1)pdm09 activity increases during the periods of low (February-May) and high (August-October) relative humidity has previously been reported and suggests that humidity extremes along with temperatures influence influenza virus survival and/or transmission in a complex way [5, 42–44].

Our findings add to the accumulating evidence that climate factors do affect the incidence of influenza in Southeast Asia. Our study showed that overall influenza positively correlated with relative humidity (up to lag 1) and rainfall (lag 1) similar to an earlier report in the tropics including a previous Thai study with data from 2009–2014 [13, 26]. One of the most significant findings from this study was that temperature, relative humidity, and rainfall were all associated with influenza A(H3N2) activity in Bangkok. Moreover, temperature was positively correlated with overall influenza activity at 4 months lag, which happens to coincide with the period of increased rainfall and humidity. Previous studies in Cambodia, the Philippines and Vietnam have linked rainfall to increased overall influenza [8, 45]. In another Philippines study, temperature and specific humidity were positive indicators for both influenza A and B viruses [2]. On the contrary, Singapore identified negative correlations between relative humidity and both influenza A and B viruses [25]. Despite differences in conclusions, we believe that findings from each of these studies are valid for their respective study sites due to intrinsic differences in geography among these countries. Seemingly contradictory results further underscore the complexity of climatic drivers on individual (sub)type of influenza, and it has been proposed that future regional data are warranted especially for relatively large and dispersed countries such as the Philippines and Indonesia [9].

The association between climate factors such as temperature with the onset of influenza epidemic in some countries may be dependent on the latitudes [42]. It has been suggested that influenza activity in countries located at low latitudes or in the tropics significantly correlated with rainfall [8]. Influenza incidence may be linked to the latitude gradients whereby countries located between 10 and 30 degrees north latitude tend to experience influenza A activity from June to November and year-round sporadic influenza B activity [2, 46]. It was postulated that the observed increase in influenza activity in the tropics during the humid and rainy season may be indirectly linked to over-crowding in the community and virus stability on fomites [47, 48]. Our observation of low overall influenza activity during hot and dry periods is consistent with the notion that increase temperature may limit aerosol transmission in tropical regions [5].

Using ARIMA model, the relationship between all influenza incidence and meteorological variables enabled a forecast model with significant prediction. The analyzed forecasts for individual influenza A(H1N1)pdm09, A(H3N2), and influenza B virus, however, were not as robust. Although factoring in the average temperature, relative humidity, and rainfalls can increase the accuracy of the forecasting model, each influenza (sub)type was affected by specific climate factors and presented forecasting challenges. Better prediction result from overall influenza activity might have been due to the increased sample size of the combined data for all three (sub)types of influenza viruses. Interestingly, we found that influenza A(H1N1)pdm09 did not always co-circulate with influenza A(H3N2) virus like it did with influenza B virus at up to 1 month lag. We also did not observe the inverse correlation between influenza A(H3N2) and influenza B virus previously identified in the tropical Uganda [14]. Again, there are probably unaccounted differences in geographical factors in addition to the fact that we did not have weekly climate data, which would have been more useful for our calculations. Finally, our time series analysis showed that the best-fit model resulted when incorporating the climate factor parameters.

This study had several limitations. Influenza surveillance was derived from one major hospital in western Bangkok and may not have represented the influenza activity of the entire city. Our surveillance was passive in nature because samples were submitted to us rather than actively sought, therefore we did not use the time of viral exposure for analysis. The overwhelming majority of the samples came from in-patient settings, so nosocomial influenza was not considered. Although we had influenza data prior to 2011, these were excluded due to fewer sample size and possible bias from the influenza pandemic of 2009. From our experience, we presumed that only influenza A(H1N1)pdm09 and A(H3N2) viruses comprised the overwhelming majority of influenza A virus in circulation, but it is possible that other less frequent subtypes were missed. Due to latitude and climate differences, influenza incidence in northern and southern Thailand may be different and this would benefit from future studies. In addition, daily or weekly climate factor values, when available, would be more useful in improving modeling accuracy. Despite these limitations, our results suggest that certain climate factors do influence influenza activity and further support the existing schedule for influenza vaccination prior to the rainy season. The time series analysis of influenza virus could potentially supplement a more accurate forecasting of future seasonal influenza activity when combined with an active epidemiological surveillance. Countries lacking the latter but share similar geographical and climatological factors may benefit from the model-based disease predictions. This and other time series analysis, especially when performed on the daily climate factors, could potentially improve the foundation for enhanced modeling of disease seasonality.

## Supporting information

S1 Fig The original and log_10_ transformed time series plots of the monthly confirmed influenza-positive cases. Original time series (left panels) and transformed time series (right panels) are shown for influenza A(H1N1)pdm09 (A and B), influenza A(H3N2) (C and D), influenza B (E and F), and all influenza viruses (G and H).
(TIF)

S2 Fig Plots of residuals of selected forecasting model, autocorrelation function (ACF), and the normal distribution of the residuals. The left column represents the residuals of univariate models, while the right column represents the residuals of multivariate models. (A and B) influenza A(H1N1)pdm09, (C and D) influenza A(H3N2), (E and F) influenza B, (G and H) all influenza viruses.
(TIF)

S3 Fig Plots of residuals of a selected forecasting model, autocorrelation function (ACF), and partial autocorrelation function (PACF). The left column represents the residuals of univariate models, while the right column represents the residuals of multivariate models. (A and B) Influenza A(H1N1)pdm09 virus. (C and D) Influenza A(H3N2) virus. (E and F) Influenza B virus. (G and H) All influenza viruses.
(TIF)

S1 Table Comparison of the mean meteorological variables between no influenza activity month and influenza-active month. (DOCX)

S2 Table Cross-correlation between individual and all influenza viruses with climate factors at different lag times. (DOCX)

S3 Table Parameters estimated by ARIMA(X) model with RMSE, AICc, MAPE, and coefficient values. (DOCX)
