# Supplementary figures and images for: Climate factors influence seasonal influenza activity in Bangkok, Thailand
Source: PLoS One. 2020 Sep 29;15(9):e0239729. doi: 10.1371/journal.pone.0239729 (PMC7523966; doi:10.1371/journal.pone.0239729)

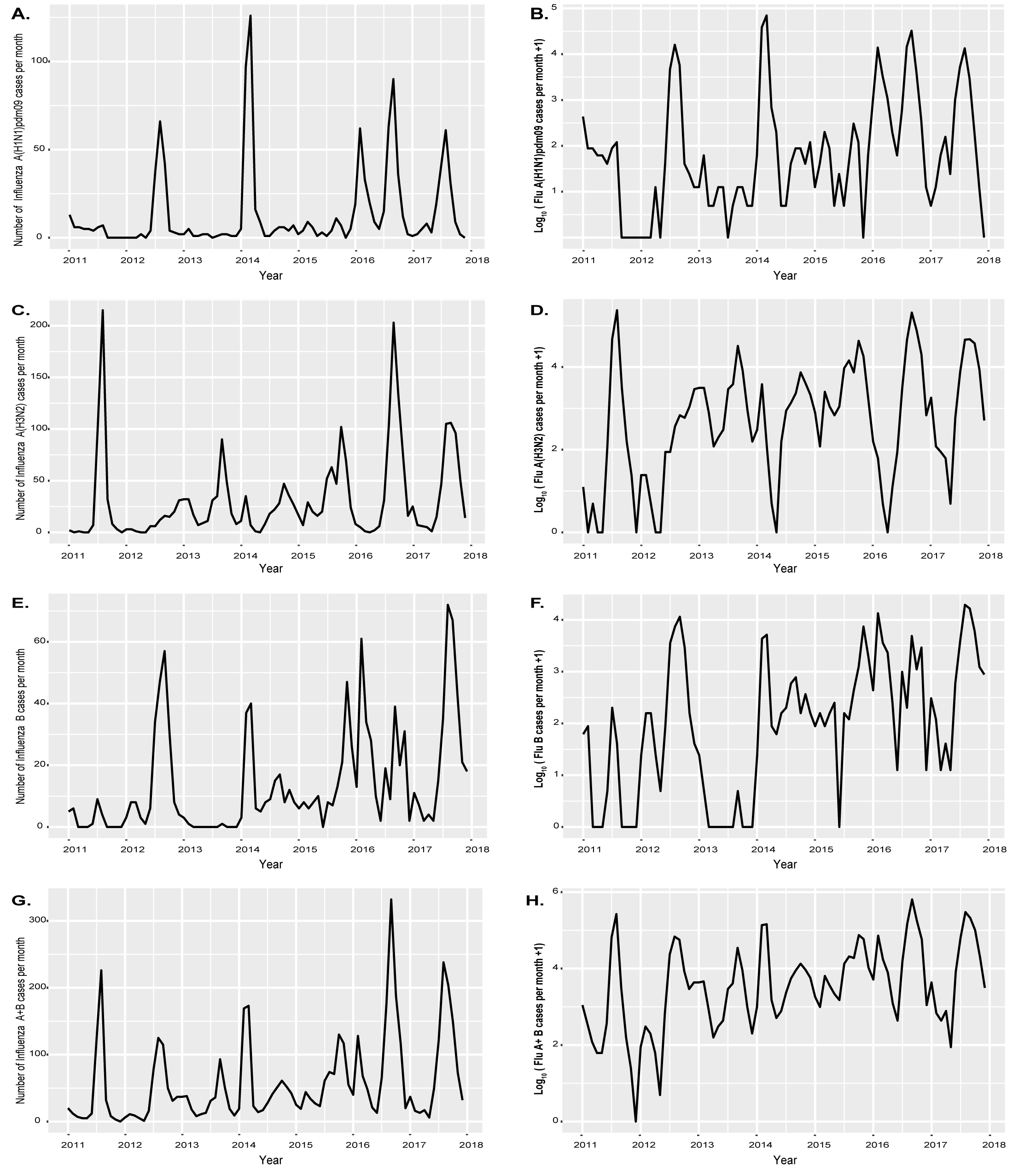

Supplement: S1 Fig — Original time series (left panels) and transformed time series (right panels) are shown for influenza A(H1N1)pdm09 (A and B), influenza A(H3N2) (C and D), influenza B (E and F), and all influenza viruses (G and H). (TIF) [file pone.0239729.s001.tif]

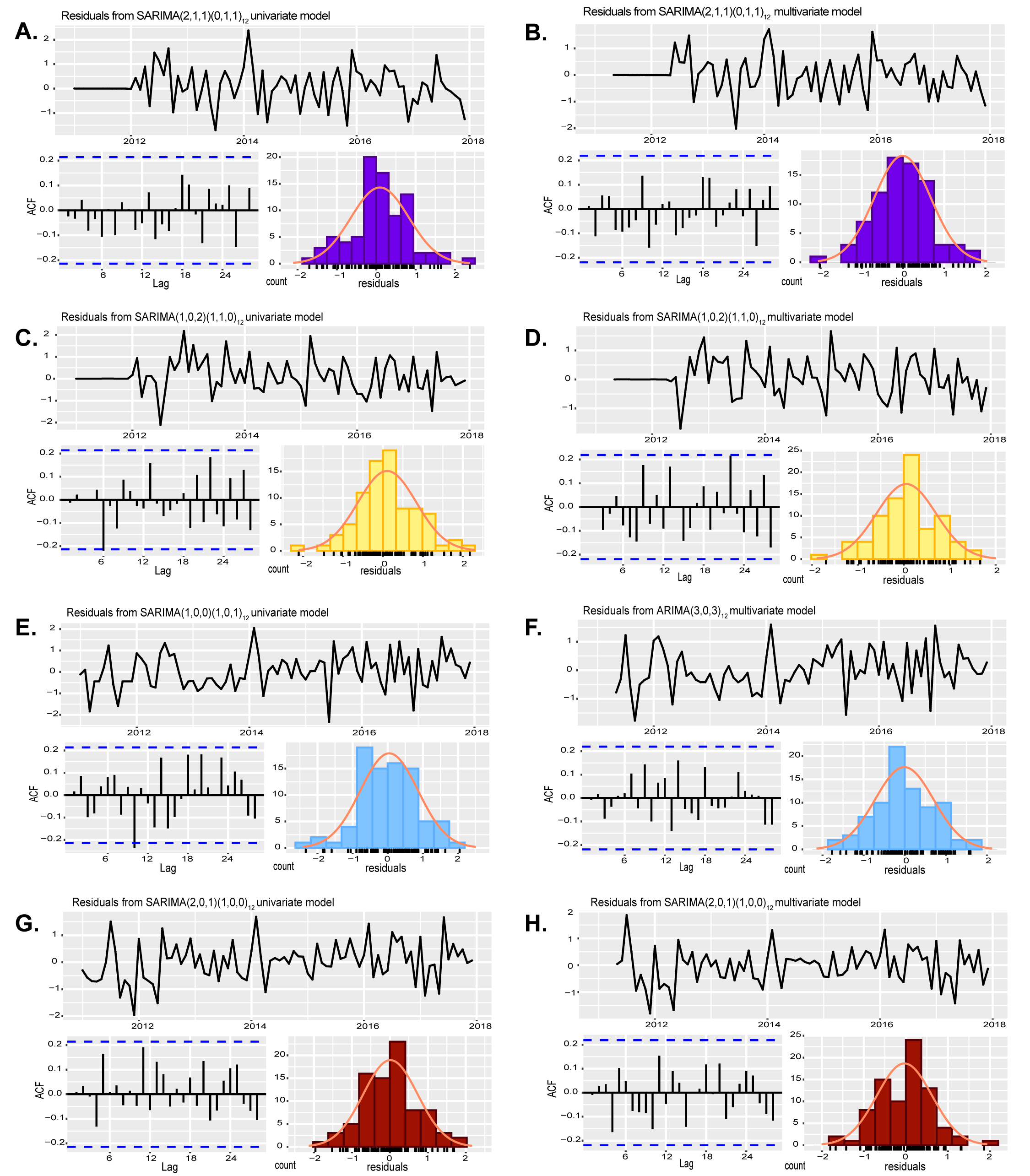

Supplement: S2 Fig — The left column represents the residuals of univariate models, while the right column represents the residuals of multivariate models. (A and B) influenza A(H1N1)pdm09, (C and D) influenza A(H3N2), (E and F) influenza B, (G and H) all influenza viruses. (TIF) [file pone.0239729.s002.tif]

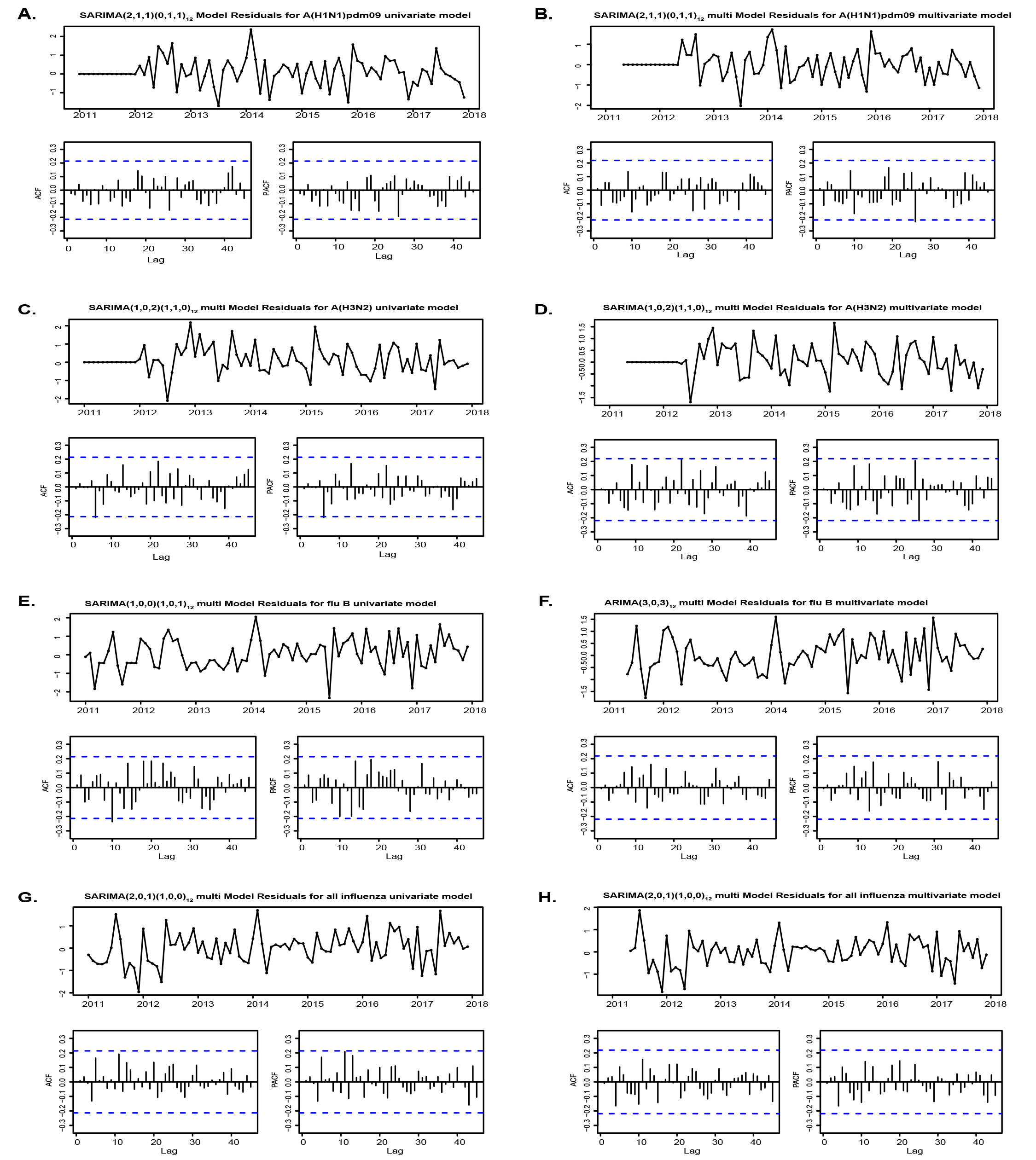

Supplement: S3 Fig — The left column represents the residuals of univariate models, while the right column represents the residuals of multivariate models. (A and B) Influenza A(H1N1)pdm09 virus. (C and D) Influenza A(H3N2) virus. (E and F) Influenza B virus. (G and H) All influenza viruses. (TIF) [file pone.0239729.s003.tif]
