# Supplementary material for: Climate factors influence seasonal influenza activity in Bangkok, Thailand
Source: PLoS One. 2020 Sep 29;15(9):e0239729. doi: 10.1371/journal.pone.0239729 (PMC7523966; doi:10.1371/journal.pone.0239729)
Supplement: S1 Table — (DOCX) [file pone.0239729.s004.docx]

**S1 Table. Comparison of the mean meteorological variables between no influenza activity month and influenza activity month.**

| **Meteorological variables** | **Mean Rank** | | **p-value** |
| --- | --- | --- | --- |
|  | **No influenza activity month^a^** | **Influenza activity month^b^** |  |
| **Mean Temperature (°C)** | 57.31 | 47.52 | 0.141 |
| **Relative humidity (%)** | 47.10 | 72.89 | <0.001*** |
| **Rainfall (cm^3^)** | 51.78 | 61.26 | 0.154 |

Asterisk denotes significance: *** p<0.001.

^a^ Defined as a monthly case proportion of <10%.

^b^ Defined as a monthly case proportion of ≥10%.
