# Supplementary material for: Climate factors influence seasonal influenza activity in Bangkok, Thailand
Source: PLoS One. 2020 Sep 29;15(9):e0239729. doi: 10.1371/journal.pone.0239729 (PMC7523966; doi:10.1371/journal.pone.0239729)
Supplement: S2 Table — (DOCX) [file pone.0239729.s005.docx]

**S2 Table. Cross-correlation between individual and all influenza viruses with climate factors at different lag times.**

| **Influenza A(H1N1)pdm09 virus** | | | | | |
| --- | --- | --- | --- | --- | --- |
|  | **Lag0** | **Lag1** | **Lag2** | **Lag3** | **Lag4** |
| **Temp** | 0.158 | 0.069 | 0.023 | 0.111 | 0.166 |
| **p-value** | 0.185 | 0.672 | 0.242 | 0.836 | 0.113 |
| **RH** | 0.118 | -0.050 | -0.182 | -0.190 | -0.198 |
| **p-value** | 0.630 | 0.513 | 0.108 | 0.103 | 0.055 |
| **RF** | 0.167 | 0.025 | -0.069 | 0.011 | -0.054 |
| **p-value** | 0.106 | 0.772 | 0.391 | 0.945 | 0.635 |
| **H3** | 0.196 | 0.040 | -0.124 | -0.210 | -0.264 |
| **p-value** | 0.054 | 0.434 | 0.450 | 0.053 | 0.018* |
| **B** | 0.633 | 0.526 | 0.294 | 0.130 | 0.099 |
| **p-value** | <0.001*** | <0.001*** | 0.024* | 0.511 | 0.566 |
| **Influenza A(H3N2) virus** | | | | | |
|  | **Lag0** | **Lag1** | **Lag2** | **Lag3** | **Lag4** |
| **Temp** | -0.228 | -0.021 | 0.306 | 0.576 | 0.646 |
| **p-value** | 0.083 | 0.567 | 0.038* | <0.001*** | <0.001*** |
| **RH** | 0.502 | 0.471 | 0.349 | 0.197 | 0.015 |
| **p-value** | <0.001*** | <0.001*** | 0.002** | 0.093 | 0.865 |
| **RF** | 0.311 | 0.436 | 0.459 | 0.467 | 0.287 |
| **p-value** | 0.036* | <0.001*** | 0.001** | <0.001*** | 0.058 |
| **H1** | 0.196 | 0.210 | 0.158 | 0.176 | 0.213 |
| **p-value** | 0.054 | 0.095 | 0.249 | 0.180 | 0.055 |
| **B** | 0.292 | 0.194 | 0.088 | -0.006 | -0.006 |
| **p-value** | 0.011* | 0.125 | 0.602 | 0.829 | 0.990 |
| **Influenza B virus** | | | | | |
|  | **Lag0** | **Lag1** | **Lag2** | **Lag3** | **Lag4** |
| **Temp** | -0.021 | -0.046 | 0.074 | 0.253 | 0.374 |
| **p-value** | 0.666 | 0.365 | 0.613 | 0.295 | 0.013* |
| **RH** | 0.152 | 0.110 | -0.030 | -0.085 | -0.096 |
| **p-value** | 0.535 | 0.656 | 0.576 | 0.365 | 0.406 |
| **RF** | 0.067 | 0.132 | 0.094 | 0.127 | 0.137 |
| **p-value** | 0.391 | 0.181 | 0.353 | 0.245 | 0.389 |
| **H1** | 0.633 | 0.585 | 0.417 | 0.219 | 0.100 |
| **p-value** | <0.001*** | <0.001*** | <0.001*** | 0.049* | 0.449 |
| **H3** | 0.292 | 0.184 | -0.002 | -0.115 | -0.100 |
| **p-value** | 0.011* | 0.114 | 0.976 | 0.296 | 0.370 |
| **all influenza viruses** | | | | | |
|  | **Lag0** | **Lag1** | **Lag2** | **Lag3** | **Lag4** |
| **Temp** | -0.132 | -0.082 | 0.162 | 0.473 | 0.617 |
| **p-value** | 0.936 | 0.417 | 0.796 | 0.005** | <0.001*** |
| **RH** | 0.392 | 0.323 | 0.128 | 0.030 | -0.045 |
| **p-value** | 0.002** | 0.006** | 0.324 | 0.970 | 0.470 |
| **RF** | 0.232 | 0.296 | 0.277 | 0.337 | 0.222 |
| **p-value** | 0.050 | 0.014* | 0.081 | 0.023* | 0.215 |

RH, Relative Humidity; RF, Rainfall; Temp, Temperature.

Asterisk denotes significance; * p<0.05, ** p<0.01, *** p<0.001.
