# Supplementary material for: Climate factors influence seasonal influenza activity in Bangkok, Thailand
Source: PLoS One. 2020 Sep 29;15(9):e0239729. doi: 10.1371/journal.pone.0239729 (PMC7523966; doi:10.1371/journal.pone.0239729)
Supplement: S3 Table — (DOCX) [file pone.0239729.s006.docx]

**S3 Table. Parameters estimated by ARIMA(X) model with RMSE, AICc, MAPE, and coefficient values.**

| **Influenza virus** | **Univariate Analysis** | | | | | **Multivariate Analysis** | | | | | | |
| --- | --- | --- | --- | --- | --- | --- | --- | --- | --- | --- | --- | --- |
|  | **Model** | **Fit** | | **Pred.** | | **Model** | **Fit** | | **Pred.** | | **Vars.** | **Coef.** |
|  |  | **RMSE** | **AICc** | **RMSE** | **MAPE** |  | **RMSE** | **AICc** | **RMSE** | **MAPE** |  |  |
| **A(H1N1)pdm09** | SARIMA(2,1,1)(0,1,1)_12_ | 0.74 | 209.06 | 1.10 | 28.63 | SARIMA(2,1,1)(0,1,1)_12_ | 0.68 | 196.84 | 0.89 | 22.99 | H1N1 (lag 1)* | -0.1021 |
|  |  |  |  |  |  |  |  |  |  |  | H3N2 (lag 4) | -0.4088 |
|  |  |  |  |  |  |  |  |  |  |  | B (lag 1) | -0.0197 |
| **A(H3N2)** | SARIMA(1,0,2)(1,1,0)_12_ | 0.73 | 187.99 | 1.33 | 40.54 | SARIMA(1,0,2)(1,1,0)_12_ | 0.65 | 176.89 | 0.77 | 22.47 | Temp (lag 4) | -0.0214 |
|  |  |  |  |  |  |  |  |  |  |  | RH (lag 1) | -0.0095 |
|  |  |  |  |  |  |  |  |  |  |  | RF (lag 1) | 0.0286 |
|  |  |  |  |  |  |  |  |  |  |  | H3N2 (lag 1)* | 0.9029 |
| **B** | SARIMA(1,0,0)(1,0,1)_12_ | 0.82 | 222.38 | 1.41 | 60.88 | ARIMA(3,0,3)_12_ | 0.71 | 203.04 | 1.14 | 46.09 | Temp (lag 4) | 0.1536 |
|  |  |  |  |  |  |  |  |  |  |  | H1N1 (lag 1) | 0.3318 |
|  |  |  |  |  |  |  |  |  |  |  | B(lag 1)* | 0.2352 |
| **All** | SARIMA(2,0,1)(1,0,0)_12_ | 0.67 | 191.25 | 0.61 | 10.93 | SARIMA(2,0,1)(1,0,0)_12_ | 0.65 | 186.27 | 0.15 | 2.81 | Temp (lag 4) | 0.0090 |
|  |  |  |  |  |  |  |  |  |  |  | RH (lag 1) | -0.0222 |
|  |  |  |  |  |  |  |  |  |  |  | RF(lag 1) | 0.0236 |
|  |  |  |  |  |  |  |  |  |  |  | All Flu (lag 1)* | 0.8726 |

Temp=Temperature, RH= Relative humidity, RF=Rainfall, All= influenza A(H1N1)pdm09+A(H3N2)+B, RMSE= Root Mean Square Error, AICc= corrected Akaike Information Criterion, MAPE= mean absolute percentage error, Pred=prediction, Coef=coefficient.

Asterisk indicates the auto-lag for each model by using the previous one month incidence to predict the next month incidence.
